# World assumptions and their role when facing trauma in urban Sierra Leone: a complementary mixed methods study

**DOI:** 10.1080/17482631.2025.2554483

**Published:** 2025-09-02

**Authors:** Andreas Steidl, Aruna Kamara, Abdul Aziz, Anthony Sheku Massaquoi, Silvia Exenberger

**Affiliations:** a Department of Psychology, University of Innsbruck, Innsbruck, Austria; b Science Department, Freetown Polytechnic, Freetown, Sierra Leone; c Department of Sociology and Social Work, University of Science and Technology, Makeni, Sierra Leone; d Department of Business Administration and Entrepreneurship, Institute of Public Administration and Management, University of Sierra Leone, Freetown, Sierra Leone; e Department of Child and Adolescent Psychiatry, Medical University of Innsbruck, Innsbruck, Austria

**Keywords:** Sierra Leone, world assumptions, trauma, mixed methods, embedded design

## Abstract

**Background and purpose:**

Concerns have been raised about the cross-cultural applicability of Western-centric models of world assumptions, questioning their universality, patterns and role when facing trauma. Within a trauma-focused research project in Sierra Leone, this sub-study examines these issues in the local context.

**Methods:**

We conducted a qualitatively driven mixed methods study (embedded design) from an emic-etic perspective. Qualitative interviews explored the assumptive worlds of 16 students, while questionnaires examining how assumptions were affected by trauma were completed by 280 students. All participants had experienced traumatic events (DSM-5). In addition, four expert interviews were conducted to address both aspects.

**Findings:**

The concept of world assumptions (benevolence, meaningfulness, self) was found to be relevant in the Sierra Leonean sample; however, the patterns differed from those typically documented in Western societies. Additional assumptions concerning the self, the world, others, and meaningfulness were identified. Quantitative results indicated that participants’ assumptive worlds were challenged by trauma, shaping their personal life stories and identities.

**Conclusions:**

To strengthen culturally sensitive investigations, we propose renaming certain domains and dimensions to more neutral and inclusive ones, and considering additional facets. This study contributes to a more nuanced understanding of world assumptions and their role in diverse cultural contexts.

## 1. Introduction

In Janoff-Bulman’s (1989) “model of basic assumptions”, the foundational elements of individuals’ assumptive worlds are identified as abstract beliefs about themselves, the external world, and the intricate connections between the two. The development of these core assumptions begins in early childhood, influenced significantly by cultural and societal factors. As individuals grow, their internal representations are shaped and reinforced, creating a shared symbolic world within their society that establishes communal expectations for daily life. These shared beliefs act as a guiding framework, helping individuals to comprehend and integrate experiences and ultimately providing a sense of meaning to their world. As individuals go through adolescence, these assumptions become generalized across various situations. The process of assimilating new information into existing schemas rather than accommodating them becomes predominant, leading these assumptions to remain largely unchanged and unquestioned throughout a person’s life (Janoff-Bulman, 1992).

### 1.1. Model of basic assumptions

Janoff-Bulman (1989) specifically distinguishes between fundamental beliefs about general benevolence (of people and the world), meaningfulness (justice, controllability, randomness), and worthiness of the self (self-worth, self-control, luck). Firstly, benevolence of the world encompasses individuals’ basic beliefs about the world’s and people’s inherent benevolence or malevolence. It addresses questions such as whether people perceive the world positively or negatively, the prevalence of good versus bad events, and whether the world referring to both people and events, is viewed as a safe place. This category also explores notions of whether individuals see others as essentially good, kind, and helpful. Secondly, the meaningfulness of the world involves people’s beliefs about the distributional principles regarding good versus bad events or outcomes. This includes considerations of action-outcome contingency, beliefs about control over one’s circumstances, and the distinction between random and reasoned occurrences. Thirdly, worthiness of the self relates to the individuals’ evaluation of their personal integrity and adherence to distributional principles at the individual level. This category examines how individuals perceive themselves, evaluating whether they view themselves as moral, worthy, and good persons and whether their behaviour, in their view, aligns with ethical and precautionary principles.

It is noteworthy that Janoff-Bulman developed her theoretical framework and the associated quantitative measurement, the World Assumption Scale (WAS; 1992), within the context of Western (USA) culture. Acknowledging potential biases, she conceded that the proposed patterns of assumptive world dimensions may reflect a positive bias inherent to Western individualistic societies. Her findings indicated a prevailing optimism among individuals in the Western context, fostering beliefs in a fundamentally good and safe world. This optimism extended to perceptions of personal goodness, competence, and morality, aligned with the “just world theory” posited by Lerner (1970), in which people believe in a world where individuals get what they deserve and deserve what they get.

### 1.2. World assumptions across cultures

It was noted that certain core assumptions guide human lives universally and have cross-cultural applicability (Splevins et al., 2010). However, numerous researchers emphasize that different cultures may harbour distinct world assumptions (e.g., Hofstede, 1980; Laungani, 1997). Examining variations in a culture’s shared assumptive worlds, Carboon et al. (2005) assert that beliefs in a just world may not be uniformly held across cultures outside the Western context. Additionally, a sense of controllability and agency may be less pronounced in non-Western cultures, as noted by Laungani (1997). Furthermore, there is a notable distinction in cognitive focus on the self between individualistic and collectivistic cultures. In individualistic cultures a person’s cognitive emphasis predominantly focuses on the self and its beliefs about the meaning of life. Collectivistic cultures tend to ground meaning in the social and cultural context of society (Bracken, 1998). These cultural variations emphasize the importance of recognizing and understanding diverse patterns of world assumptions when studying human behaviour and cognition across different cultural contexts.

### 1.3. Changes of the assumptive worlds through trauma

While the alteration of the most fundamental beliefs is considered rare and less likely to occur over time (Janoff-Bulman, 1992), there are instances in adulthood where basic assumptions may undergo modification. The “Shattered Assumptions Theory” (Janoff-Bulman, 1992) states that traumatic events can present data that contradict, seriously challenge, or shatter pre-existing assumptions and lead to mental health problems. This theory is frequently referred to in social-cognitive trauma research. Researchers reported traumatic experiences to have affected the world assumptions of individuals in the long run, both in Western (e.g., USA: Kaler, 2009; Netherlands: Giesen-Bloo & Arntz, 2005) and in non-Western cultures (South Africa: Magwaza, 1999; Kenya: Ferrajão et al., 2023) by using the WAS. However, there are mixed findings regarding the construct validity of the instrument. Some studies have questioned its validity (e.g., Jeavons & Godber, 2005; Kaler et al., 2008), while others have successfully replicated the eight proposed factors in Western populations (Elklit et al., 2007; van Bruggen et al., 2018).

Research on world assumptions in non-Western cultures is scarce, particularly studies not relying on the WAS. A review of the literature revealed only one emic study—a qualitative one—focusing on the assumptive worlds of unaccompanied refugee minors from war-torn Syria and Afghanistan. While the study identified statements aligning with Janoff-Bulman’s dimensions, it also introduced a meaningfulness subdomain, labelled the “principle of a metaphysical plan” by referring to a belief in a higher plan, destiny, or the will of God (Gottschald & Sierau, 2020). This underlines the need for more comprehensive investigations into world assumptions in diverse cultural contexts, utilizing culturally sensitive methodologies.

### 1.5. Shattered world assumptions and posttraumatic growth across cultures

According to Janoff-Bulman (1992), individuals who have experienced trauma face the task of rebuilding their shattered assumptions in the aftermath of such an event. The process involves re-establishing, reconstructing and adapting their assumptive worlds to integrate new trauma-related information, and ultimately aid in coping and recovery. The posttraumatic growth (PTG) model, developed by Tedeschi and Calhoun (1996) and expanded by Tedeschi et al. (2018), builds on Janoff-Bulman’s “Shattered Assumptions Theory”. In this model, the restoration of the assumptive worlds is considered a prerequisite for achieving growth. While cultural influences are acknowledged, it maintains cultural neutrality regarding the nature of these core beliefs. Splevins et al. (2010) warned that presuming specific and universal patterns of core beliefs may introduce cultural biases. They furthermore emphasized the potential for cross-cultural variations in the impact of trauma on worldviews. In 2016 Winter et al. highlighted in their qualitative studies in Sierra Leone that, unlike assumptions in the West, individuals in crisis-ridden countries like Sierra Leone consider trauma, victimization, and adversity as inherent aspects of life rather than exceptionally “shattering” experiences. In Sierra Leone and similar contexts, where collective traumas (e.g., prolonged civil conflict, the Ebola epidemic, and recently the Covid-19 crisis) are embedded in the societal fabric, individuals may not have schematic expectations that adversity or traumatic experiences are unlikely to occur.

In Sierra Leone Steidl et al. (2024) replicated a validation study of the PTG and posttraumatic depreciation (PTD) theoretical model across ten countries and included theory-driven variables, such as re-examination of core beliefs and event centrality (Taku et al., 2021). Varied findings in these variables were reported across and within individualistic and collectivistic countries by Taku et al. (2021). The etic approach did not include an examination of the diverse natures of world assumptions across cultures. However, for Sierra Leone, Steidl et al. (2024) reported that the re-examination of core beliefs and event centrality play a significant and predictive role for both PTG and PTD. Although the study also did not explore the patterns of Sierra Leoneans’ world assumptions, it indicated that traumatic experiences—which people there might regard as an inevitable part of daily life—still affect their assumptive worlds and sense identity. A further investigation into the Sierra Leonean assumptive worlds and their presumably complex role when facing trauma is therefore necessary.

### 1.5. The present study

This comprehensive mixed methods study seeks to thoroughly explore and specify the diverse dimensions of world assumptions among Sierra Leonean tertiary education students who have previously experienced a DSM-5 traumatic event (Criterion A for PTSD; American Psychiatric Association, 2013). It will also conduct an in-depth investigation into how these traumatic experiences affect the dimensions of their assumptive worlds. In other words, it aims at answering two key questions, namely: What assumptions do Sierra Leonean students have about themselves, others, the external world, and the events occurring in it? Which assumptions were affected by a traumatic event and to what extent?

The qualitative component of the research delves deeply into the current world assumptions of Sierra Leonean students. The aim is to provide a nuanced understanding of the intricacies of their assumptive worlds. Quantitatively the study aims at offering insights into the applicability and structure of the “Shattered Assumptions Theory” (Janoff-Bulman, 1992) within an urban Sierra Leonean student context. Additionally, it explores potential differences related to sex and religion (Muslims vs. Christians) and examines the relationship between elapsed time since the traumatic event and the present age. Expert interviews are conducted to gain culture-specific insights into both topics. The integration of both types of data addresses previously unexplored questions from a culture-sensitive perspective, considering emic as well as etic viewpoints. The findings will contribute to a deeper understanding of the interplay between traumatic experiences and world assumptions in a specific cultural context.

## 2. Method

### 2.1. Emic-etic approach

Etic research approaches phenomena from the perspective of the external observer, while emic research seeks to understand them from the perspective of the subjects themselves (Beals et al., 2020). The research team blends insider (i.e., emic) and outsider (i.e., etic) perspectives. Three investigators are lifelong residents of the participant catchment area; their scholarly training is complemented by lived experience in the local culture. Two additional scholars—specialists in trauma and posttraumatic changes—approach the project from a Western background, supplying an external vantage point. This mix of positionalities has enabled us to interpret psychological processes through culturally grounded theories and frameworks (Yang, 2000) while remaining alert to the pitfalls of importing concepts wholesale from other contexts (Tweed & DeLongis, 2006). From study design through data analysis and interpretation, we practiced continual reflexivity, carefully keeping the balance between both emic and etic perspectives. The emic-etic approach extended to the applied measures and concepts. Comprehensive semi-structured interview guides were developed together by researchers of both origins; questionnaires developed by Westerners were applied after backtranslation to Sierra Leonean English.

### 2.2. Research design

We opted for a qualitatively driven, concurrent, and embedded mixed methods design, denoted as QUAL(Quan). The core component involved qualitative interviews (QUAL) to address the open research question and capture an emic understanding of Sierra Leonean student world assumptions, going beyond the “model of basic assumptions” proposed by Janoff-Bulman (1989). As a supplemental and embedded component, we conducted a quantitative (Quan) investigation to assess the re-examination of core beliefs and event centrality after a traumatic event, using a larger and more representative student sample.

**Figure 1.**
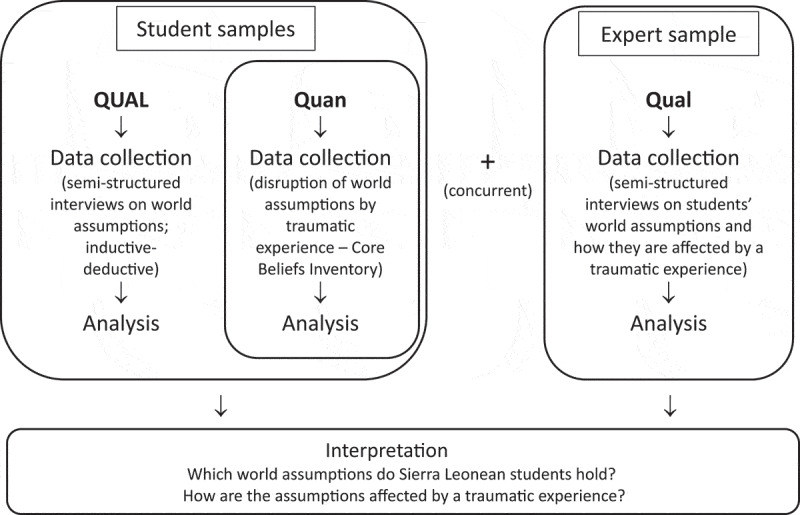
Flowchart of the study’s expanded concurrent embedded mixed methods design.

During the initial stages of the study, we realized the insufficiency of our design in capturing a comprehensive understanding, due to the open-ended research questions and diverse statements from students. Additionally, while searching for relevant literature in university libraries across Freetown, we noted a lack of research conducted on the topic within Sierra Leone itself. Therefore, we introduced another supplemental qualitative (Qual) component involving expert interviews to support both QUAL and Quan data. Data from QUAL, Quan, and Qual components were collected concurrently, analysed independently, and subsequently integrated during the discussion stage. The visual representation of our study design, QUAL(Quan) + Qual, is illustrated in Figure 1.

### 2.3. Participants

Given that 80% of the country’s population is aged 35 and below (IIEP-UNESCO Dakar, 2020), our study exclusively focuses on students, recognizing that they represent a subset of the youthful population. Inclusion criteria for both qualitative and quantitative samples required participants to be at least 18 years old, proficient in English, Sierra Leone’s official language, and to have a Sierra Leonean cultural background. Detailed socio-demographic data of the samples is presented in Table 1.

**Table 1.** Socio-demographic data of the two student samples (QUAL *N* = 16; Quan *N* = 280).

	Demographics	QUAL *n (%)*	Quan *n (%)*
Sex	male	7 (43.8)	147 (52.5)
	female	9 (56.3)	133 (47.5)
Religion	Muslim	8 (50)	141 (48.9)
	Christian	8 (50)	137 (50.4)
	Muslim & Christian	0	1 (0.4)
	Jehovah Witness	0	1 (0.4)
	active practice Religion	15 (93.8)	256 (95.2)
	no active practice Religion	1 (6.3)	11 (3.9)
	no information	0	13 (4.6)
		***M (SD; R)***	***M (SD; R)***
Age	male	29.14 (7.5; 23–42)	26.61 (6.5; 18–63)
	female	30.11 (5.9; 20–37)	28.71 (9.2; 18–58)
	total	29.69 (6.2; 20–42)	27.61 (8.0; 18–63)
	Age at event (years)	19.74 (6.8; 11–34)	21.29 (8.0; 2–54)
	elapsed time since event (years)	9.95 (8.2; 0.3–23)	6.32 (7.7; 0.1–35)

*M* = Mean; *SD* = Standard Deviation; *R* = Range.

#### 2.3.1. Participants of the qualitative study

*Student interviewees*. A non-clinical sample of 17 students (9 women and 8 men) from various departments (Economics, Political Science, Education, Home Economics) at Freetown Polytechnic was recruited by the first author between November 2022 and February 2023. They participated voluntarily in the qualitative study on their current assumptive worlds. We focused on participants who experienced a DSM-5 traumatic event. Due to language problems, one interview had to be excluded. The remaining 16 interviewees (9 women and 7 men) had a mean age of 29.69 years (age range 20–42), with a traumatic event occurring 9.95 years in the past on average (range 0.3–23). All 16 students were also part of the quantitative sample after having answered the questionnaires before the interviews.

*Expert participants*. Four experts, including two lecturers (one female and one male) from the Peace and Conflict Department at Fourah Bay College in Freetown, a male Psychology lecturer at Milton Margai University in Freetown, and a male lecturer in Sociology and Social Work at the University of Makeni, residing in Freetown, were interviewed. These professionals, engaged by the first author, had expertise in fields related to our research questions and voluntarily participated without any incentive.

#### 2.3.2. Participants of the quantitative study

A non-clinical sample of 310 students from the four tertiary education institutions in Sierra Leone (Technical, College, Polytechnic, University) was randomly recruited between November 2022 and February 2023 in Freetown by the first and second authors. Participants were studying in various departments (e.g., Human Resources, Economics, Home Economics, Political Science, Sociology and Social Work, Education). Only participants reporting a DSM-5 traumatic event were included in the data analysis, resulting in 280 participants (63 Technical, 75 College, 71 Polytechnic, 71 University). The mean age was 27.61 years (range 18–63), with a mean time of 6.32 years (range 0.1–35) since the reported traumatic experience.

Both the qualitative and quantitative student samples were almost evenly divided regarding sex and religion. Over 90% of the participants indicated active religious practice, regardless of whether they were Muslim or Christian.

### 2.4. Procedure

Prior to participating in either the quantitative or qualitative study, all participants gave verbal and written informed consent. Participation was voluntary, with the option to withdraw from the study at any point, and anonymity was assured. No incentives were offered or provided during the course of the study. Before fieldwork commenced, the quantitative instruments were subjected to a rigorous back-translation procedure. The second author partnered with a Sierra Leonean university lecturer in English to render the original questionnaires into local English. A pivotal lexical shift replaced the words “traumatic” and “worst” with “tragic,” the term most used in Sierra Leone to denote intensely stressful events. Participants facing difficulties with questionnaire completion (paper-pencil) had the option to seek assistance from the first or second author. All student interviews took place in the same quiet and private location within the Freetown Polytechnic campus, conducted face-to-face by the first author and audio recorded. Participants in both qualitative and quantitative parts of the study were informed that, if negative feelings or emotions arose because of their participation, they could contact the first author, a trained psychologist who resided in Sierra Leone for an extended period. The experts, aware that their inclusion in the study was due to the lack of research on Sierra Leonean assumptive worlds both inside and outside their country, were interviewed in different undisturbed locations in Freetown by the first author. Verbal and written consent was obtained from each expert. Interviews with students and experts were conducted until data saturation was reached.

To ensure comprehensibility for the analysis, every interview conducted in this study was transcribed verbatim by the third and fourth authors, both Sierra Leoneans, for the benefit of the first and fifth authors, who are Austrians and conducted the analysis. This process aimed at maintaining accuracy and cultural sensitivity throughout the research.

This study was a sub-study of a research project in Sierra Leone. It received initial approval from the University of Innsbruck, Austria (Certificate of good standing 67/2002), followed by ethical clearance by Fourah Bay College (University of Sierra Leone) and Freetown Polytechnic. Other participating institutions (Freetown Murialdo and Milton Margai University in Freetown) granted permission for the survey. The entire research process adhered to the principles outlined in the Declaration of Helsinki (1964), its later amendments, and APA ethical standards (American Psychological Association, 2017).

### 2.5. Measures

#### 2.5.1. Problem‐centred interview (Witzel, 2000)

Following the inductive-deductive approach of problem-centred interviews (Witzel, 2000), the first, second, and fourth authors developed a semi-structured interview guide for the student sample. Anchoring questions were based on Janoff-Bulman’s (1989) theory of people’s basic assumptions such as: “What do you think about the world in terms of justice and fairness?” or “Why do good or bad things happen to us?” This guide helped the participants with their narratives about their assumptive worlds and ensured comparability across interviews during analysis. Additional questions were included to gain insight into how Sierra Leoneans conceptualize the self. A priori questions about participants’ outlook on their chances and future were incorporated, because of the challenging socio-economic context of Sierra Leone. These aspects were also addressed in the quantitative sample’s questionnaire. Participants were further asked about how the history and the frequent occurrence of traumatic experiences had influenced their perspective on facing adversity. Open-ended questions were integrated into the interview guide to capture potential unknown world assumptions of the sample. If needed, supplementary questions were asked for clarification at any point during the interview. As a series of topics was covered in the interview, participants also shared details about their personal most “tragic” (i.e., traumatic) events.

For the expert sample another semi-structured interview guide with open questions was created by the first, second, and fourth authors to gather their opinions and explanations regarding the world assumptions of members of their culture, particularly of students. The experts were also queried about the impact of trauma on the assumptive worlds of Sierra Leoneans. The interview guide provided a structured, yet flexible, framework for obtaining rich and nuanced insights from the experts.

#### 2.5.2. Critical life event and DSM-5 trauma Criterion A

In the quantitative sample participants were initially encouraged to provide a brief, written account of the most *tragic* event they had experienced in their lives and how long ago it had happened. Subsequently, they were asked to assess whether the mentioned event met the DSM-5 trauma criteria by responding to the following questions: (1) Did it involve actual or threatened death, serious injury, or sexual violence? (2) Did you directly experience it, witness it, hear about it happening to a close family member or close friend, or were you repeatedly exposed to it? (3) In the case of the death of a close family member or close friend, was it due to an accident, violence, or a natural cause? A traumatic event, according to DSM-5 criteria (American Psychiatric Association, 2013), was confirmed if at least one statement from both questions (1) and (2) was affirmed and, in the case of question (3), the death of a close person was due to an accident or violence. This systematic approach ensured a standardized classification of traumatic events, enhancing the reliability and consistency of the data.

#### 2.5.3. Core beliefs

The Core Beliefs Inventory (CBI; Cann et al., 2010) measures the degree to which a stressful experience led participants to examine their core beliefs on 9 items, using a 6-point Likert scale (0 = *Not at all*, 1 = *Very small degree*, 2 = *Small degree*, 3 = *Moderate degree*, 4 = *Great degree*, 5 = *Very great degree*). The Cronbach’s alpha of the CBI in our sample was 0.76.

#### 2.5.4. Event centrality

The Centrality of Event Scale (CES; Berntsen & Rubin, 2006) assesses the degree to which a referred traumatic experience was central to the participant’s life story and identity. The CES consists of 7 items, using a 5-point Likert scale (1 = *totally disagree*, 2 = *A little bit*, 3 = *Moderately*, 4 = *Quite a bit*, 5 = *totally agree*). Its Cronbach’s alpha was 0.75 in our sample.

#### 2.5.5. Demographic characteristics

Participants indicated their age, sex, religious affiliation and if they were actively practicing their religion.

### 2.6. Data analysis

#### 2.6.1. Qualitative data

We employed *reflexive* Thematic Analysis (TA, Braun & Clarke, 2019) using MAXQDA software, Version 22 to analyse the interviews. TA is well suited for our emic-etic approach, because it can be used for both inductive (data-driven) and deductive (theory-driven) analyses and can capture both explicit (manifest) and underlying (latent) meaning in qualitative data (Clarke et al., 2015). In *reflexive* TA researchers constantly reflect on subjectivity and meaning making throughout the analytic process (Braun & Clarke, 2019). Firstly, during analysis, the first and fifth authors, both experienced in cultural psychology research, were constantly mindful of their perspective as outsiders, who grew up in a Western society. Secondly, reflection focused on rigorous discussions between study authors about both etic (first and fifth authors) as well as emic (second and third authors) perspectives to prevent potential biases in analysing the interview data. Consequently, we deliberately limited the interpretative analysis of interviews and focused on staying close to participants’ original expressions. We prioritized accurate representation over imposed interpretation. Regarding the theme establishment and their naming, we preferred the proposed domains and dimensions of Janoff-Bulman (1989) than abstracting them. By doing that, a better comparison of our findings as well as more accurate implications for future cross-cultural investigations in world assumptions were facilitated.

Throughout analysis we adhered to the six phases of *reflexive* TA to analyse each interview sample separately. The first and last authors simultaneously yet independently (1) read and reread the transcripts to familiarize themselves with the content, (2) developed initial codes, either for phrases or sentences, (3) combined similar codes to corresponding themes and (4) reviewed and reassembled them into more distinct ones. Subsequently, the first and last authors (5) compared the content of their identified themes, discussing any differences in their code system until consensus was reached. At this juncture the second and third authors, both Sierra Leoneans, critically reviewed the alignment of codes to the final themes to ensure cultural sensitivity. This iterative process led to further code and theme refinement including the establishing of higher-order themes until coming to agreements. (6) Finally, the analysis was concluded and the findings were reported.

#### 2.6.2. Quantitative data

After retrieving descriptive statistics, bivariate analyses were conducted to examine relationships between the measurement totals (CBI and CES) as well as between the totals and (i) descriptive factors related to the reported trauma (Pearson’s correlation coefficients) and to compare data regarding (ii) sex and (iii) religion (independent and paired t-tests with alpha levels of .05). Statistical analyses were performed using IBM SPSS software, Version 29.

## 3. Results

### 3.1. Nature of event

A broad range of traumatic events was reported by the samples, as depicted in Table 2.

**Table 2.** Distribution of traumatic events of the samples, divided by sex and as totals.

Traumatic event	QUAL (*N* = 16)			Quan (*N* = 280)		
	*Women* (%)	*Men* (%)	*Total* (%)	*Women* (%)	*Men* (%)	*Total* (%)
Mudslides, floodings, fires	1 (11.1)	4 (57.1)	5 (31.2)	24 (18)	53 (36.1)	77 (27.5)
Accidents (e.g., road)	3 (33.3)	–	3 (18.7)	36 (27.1)	30 (20.4)	66 (23.6)
Civil war	1 (11.1)	2 (28.6)	3 (18.7)	13 (9.8)	13 (8.8)	26 (9.3)
Sexual violence	2 (22.2)	–	2 (12.5)	18 (13.5)	8 (5.4)	26 (9.3)
Physical & mental health	–	–	–	11 (8.3)	14 (9.5)	25 (8.9)
Ebola	–	1 (14.3)	1 (6.2)	10 (7.5)	14 (9.5)	24 (8.6)
Death (e.g., sudden death of a loved one)	1 (11.1)	–	1 (6.2)	10 (7.5)	10 (6.8)	20 (7.1)
Rape	1 (11.1)	–	1 (6.2)	9 (6.8)	–	9 (3.2)
Riot	–	–	–	2 (1.5)	5 (3.4)	7 (2.5)

### 3.2. Qualitative findings

We identified three domains, three subdomains (i.e., higher-order themes) and ten dimensions (i.e., themes) representing the world assumptions within our Sierra Leonean sample. For an overview see Table 3. Results of the student participant (p.) and the supplementing expert (e.) interviews are presented simultaneously, in accordance with the defined domain and dimension. All participant quotations are reproduced verbatim, preserving their original wording.

**Table 3.** Domains, subdomains, and dimension of world assumptions in our sample.

Domain	Subdomain	Dimension
Worthiness of the Self	Self-Concept	Self-Worth
		Self-Control
		Luck
		Chances/Future
Character of the world and people	Global, societal & communal levels	Character of the world
		Character of people
Meaningfulness of the world	Religiosity	Justice
		Controllability
		Reason
		Adversity

#### 3.2.1. Assumptions about the worthiness of the self

**Figure 2.**
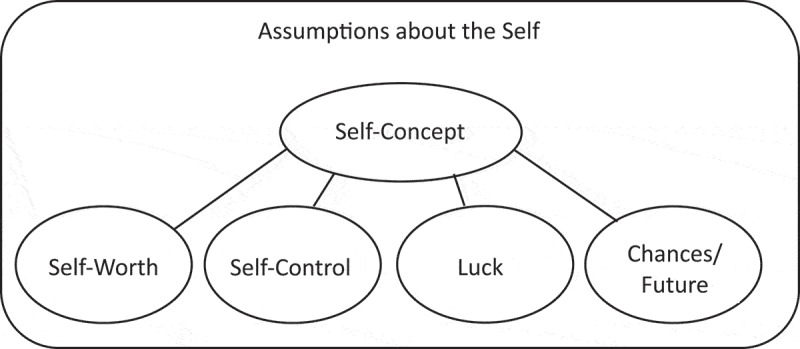
Proposed cross-cultural approach for the assumptions about the self-domain.

As a requirement for investigating the world assumptions of individuals in Sierra Leone we tried to get an understanding of how the self is conceptualized in a presumably collectivistic Sierra Leonean context (Hofstede, 2023), as presented in Figure 2. However, this difficult question was soon eliminated from the student interview guide after a few interviews because it turned out to be too wide. Likewise, other prepared indirect questions to capture the understanding of the self by asking about the relationship to their close ones did not result in sufficient answers.

Yet the experts could give insights that Sierra Leoneans do not exclude their own person to make sense of their world:

There is a general conception about unity and togetherness, but every Sierra Leonean places himself first whenever the opportunity arises. “Everybody for himself” is a popular notion in the populace…. It [collectivism] is a genetic description that disguises the reality. (e. 3)

Nevertheless, they consistently referred to Sierra Leonean society as being collectivistic:

You don’t depend on other people for everything, but the extent of dependence is very high in our society due to poverty. So, interdependence is part of our culture…. People are more helpful in the provinces than in Freetown. Individualism is predominant in Freetown. (e. 3)

Sierra Leone is a collectivistic society. In our closer relationships and communities, we help each other a lot. We depend on each other. You trust those ones that are closer to you. But there’s jealousy over influence as well. In Africa it is not easy. (e. 2)

##### Self-Worth

Statements of self-worth, self-confidence, and satisfaction were included in this dimension. 15 participants (93.75%) regarded themselves as worthy persons (e.g., p.17: “I see myself as a great lady … I never see anything negative about me”), with most of them further reporting to be satisfied with themselves. Two interviewees expressed only partial satisfaction; a third woman offered a balanced appraisal that highlighted strengths as well as areas for improvement, and five others acknowledged their own shortcomings.

Asked about the origin of the high self-esteem of students, expert 2 mentioned, “These are students—they think they will be somebody in the future.” Expert 3 likewise affirmed, “Several sources—some are sponsored and empowered by their homes.” Expert 1 more generally referred to Sierra Leoneans as having a positive mindset:

From the belief and understanding we have – we are strong people, resilient people. We kind of say: “Oh, we survived the war, so Ebola cannot kill us nor Corona.” People die, but still, we have a group of Sierra Leoneans who stay and fight on. So, I think it is that feeling of being strong, resilient, to withstand all these shocks that is responsible for it [peoples’ positivity], the energy and enthusiasm about dealing with issues.

##### Self-Control

The second dimension in the assumptions about the self-domain comprised the participants’ disclosures about self-regulation. Eleven participants (68.75%) communicated that they have control over themselves (e.g., p.6: “I won’t be moved so I don’t move on certain things”); one male student revealed that he tries to; one female student disclosed that the control over herself was not too strong, and another interviewee admitted having no control over herself. Two participants did not specify.

##### Luck

The dimension “Luck” represented the students’ opinions about their own fortune. 14 participants (87.5%) considered themselves lucky persons (e.g., p. 9: “I’m looking lucky compared to other stories—I have children, I have husband, I have my family, I’m still OK, I still fit in the society.”) One participant refrained from calling himself fortunate, noting that others appeared to be enjoying greater success, while another provided no personal evaluation at all.

##### Chances/Future

In this self-relevant dimension representations of one’s own freedom of action were captured. Ten participants (62.5%) affirmed having had chances in life and to looked positively into future (e.g., p. 6: “Every day that I wake up I think I have more than 10,000 chances to make it in life.”) Three expressed having a lot of chances because of the Grace of God (e.g., p. 9: “The first chance is I’m still alive yes thank God I’m still alive, that is a bigger chance for me compared to those who die with the same problem. I [can] see, and I’m married, I have children.”) Only two students described their prospects as limited and expressed a pessimistic view of the future, whereas one respondent offered no opinion.

We asked the experts about the optimism of Sierra Leonean students and received answers like, “They come from organized and well-off homes, can boast of good relatives that earn from their education and are independent. These serve as motivations for the students with the optimism that they will replicate those achievements” (e. 3). Expert 1 described a general optimism among the population, “Sierra Leoneans are people full of hope in the midst of our many challenges. We have more challenges than opportunities in the country, but we always believe some magic transformation will take place.”

#### 3.2.2. Assumptions about the character of the world and people (cf. benevolence)

##### Character of the world (cf. Benevolence of the world)

The participants’ appraisals of their broader, impersonal environment as benevolent or malevolent are reflected in this dimension. None of the participants referred to the world/Sierra Leone/Freetown or their living environment as a safe place. Ten participants (62.5%) expressed having a negative outlook with more evil than good things happening (e.g., p. 17: “The world is a place of struggle.”) Four participants reported seeing more good than bad events occurring in the world, yet all of them mentioned at least one negative aspect despite their positive views (e.g., but the world/Sierra Leone is “not in good shape”, “not safe”, or “wicked”). Some participants referred to “their world”, mentioning that their lives were very hard and full of obstacles. Two participants saw all environmental aspects as balanced.

##### Character of people (cf. Benevolence of people)

In this dimension focusing on the benevolence or malevolence of people, six students (37.5%) explicitly saw more evil than good people in Sierra Leone (e.g., p. 17: “This is a country in which somebody can show you their white teeth and then slap you at the back”), while nine participants referred their statements on humanity in general. Four students (25%) stated that there were more good than bad people in this world. Five (31.25%) perceived it as balanced (e.g., p. 11: “Well not everybody is perfect, some are good some are bad so let me say that one is equal.”) One participant did not clearly specify either on the world or on Sierra Leone. Eight participants (50%) explicitly pointed out that they did not fully trust or rely on any other person, for example participant 21: “I don’t trust people even 50%. I trust only in God … . As the Rastafarians say, ‘your best friend can be your secret enemy’ – so I don’t trust individuals.” Two participants stated they generally trusted people; another three mentioned they only trusted their family and close ones.

All experts affirmed that Sierra Leoneans generally lack trust in each other, as expert 4 said: “People are not trusting completely. Except their own relatives, their close relatives they may trust them, but they do not have too much trust in other people. And even total trust is never there.” Expert 1 gave a possible explanation that the mistrust, especially on a societal level, could stem from past conflictual experiences:

We have had different scenarios or incidents where Sierra Leoneans did not trust each other. For example, during the war … when the rebels came – some people joined the rebels, and they were showing [them] where the people were hiding their stuff. So, there was this feeling of betrayal, lack of trust and all of this.

##### Global, societal & communal levels

**Figure 3.**
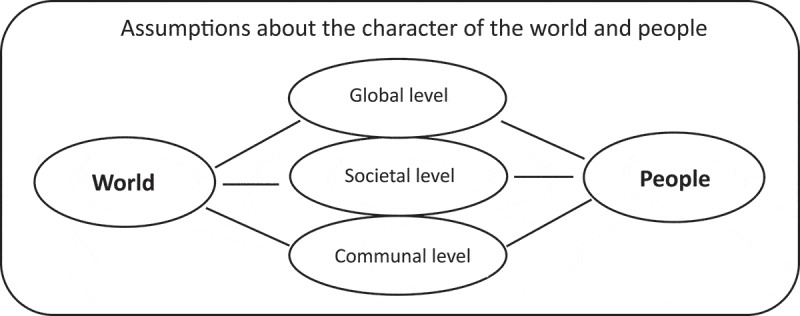
Assumptions about the character of the world and people on different levels.

Because we found that participants referred their answers to general questions about their outlook on the world and other people to different levels (e.g., the entire world, Sierra Leone, Freetown, their community, or family) and their statements sometimes varied across those levels, we created a subdomain labelled “Global, societal & communal levels” as depicted in Figure 3.

#### 3.2.3. Assumptions about meaningfulness

##### Justice

The participants’ opinions on equity were captured in this dimension. 13 participants (81.25%) negated the existence of justice and/or fairness (e.g., p. 1: “Injustice is the order of the day in Sierra Leone.”) Explaining further, some of them referred to the lack of justice either in a religious context (e.g., p. 8: “Only people that have the fear of God in them are the people that live in fairness or do justice”) or in an economic and a political context, such as participant 9: “In Sierra Leone, if you don’t have money or you are not a politician, there’s nothing like justice.” Two participants reported seeing Sierra Leone and the world as a just place.

Expert 2 stated: “They [the students] don’t think about justice. They don’t think there’s fairness in this country.” Expert 3 further differentiated:

There’s a divided notion and opinion about justice and fairness here. Usually, people in positions of power and privilege would claim everything positive in their favor, the underprivileged would experience the disadvantages and lack of justice in the society. A lot of people are poor. We are poor people.

##### Controllability

This dimension involved the student’s opinions on the extent they could influence the course of events. Nine participants (56.25%) thought that things could be controlled (e.g., “by behaviour”, “actions”, “mindset”, or “if time goes by”). Four participants said that things can be controlled to some extent, whereby two of them explicitly mentioned that, ultimately, destiny decides (e.g., p. 15: “Some we can, others we cannot. Some things are destined to happen to us. So, if you are destined to be someone, nothing and no one can stop it.”) Two participants neglected a notion of controllability and two more did not specify on this aspect.

##### Reason (cf. Randomness)

The content of this dimension focused on the causes of events. Only one person said that things happen at random and without any reason. Three participants stated that the reason why things happen in life is “due to behaviour”, with two of them specifying that bad things occurred because people “do not listen to advice”. Ten students (62.5%) expressed that the reason for events lay in destiny given by God (e.g., p. 19: “What has been destined for you is going to happen to you”; p. 11: “Anything that happens has a reason and this reason is God.”) Four of them were sure that destiny was influenceable (e.g., “by prayer” and “right actions”) or that sometimes the reason was destiny and, at other times, behaviour. One participant saw the reason for bad things was due to disobeying God and another participant did not specify.

The experts’ opinions on randomness and controllability were consistently described as religiously inclined:

People believe in destiny as a design, and at one point in life you have to fit yourself into that destiny. But it will depend on your activities and the things you get involved in. You can influence it a bit as a kind of control or intervention. (e. 2)

By believing in destiny means that you give up your control in the first place. You attribute everything that happens to the will, power, and permission of God. … People think it should be accepted as it comes, and even respected. … Sometimes we are even obliged as Muslims to thank Allah for everything that happens, whether we like it or not. (e. 3)

##### Adversity

An additional dimension for views on adverse experiences was established. Ten participants (62.5%) responded with statements referring to adversity belonging to life in Sierra Leone, e.g., participant 2: “Well in Sierra Leone we see stressful moments as part of life and … somebody saying, ‘I am stressed’ – nobody will ask you—‘Why are you stressed? What’s your problem?’ … We just see it as normal among us.” Six participants primarily talked about the negative effects such as poverty and harsh living conditions, when responding to the question on the effects of the frequent occurrence of traumatic events in Sierra Leone.

When asked how people manage to integrate adversity into their assumptive worlds, participant 12 provided a religious insight: “It will take the grace of God for you to overcome all those evil circumstances that come into your way.” Similarly, participant 6 stated: “As long as the creator is there, he can listen to your cry, so you have to move on and bulk up.” Others referred to cultural aspects (e.g., p. 2: “I don’t see it different from my culture because the culture we grow up from trained us that we should allow stressful moments to … we should let them go.”)

The experts confirmed traumatic situations to be inherent in life in the country: “Well, when you talk about these disasters, and in Sierra Leone we have lots of them, you move around the place—you know this place is hazardous” (e. 1). Or expert 3: “There is lots of people going through traumatic situations, it is not so taken serious here. … The religious people say—‘It was meant to happen’ – so that should be part of life. You should take it and move on.” When asked if there were varying interpretations of trauma in different religions, expert 4 stated: “Trauma in different religions? I think the religious people agree on that point—‘It is God’s will’ – the Muslims and the Christians agree on that.”

##### Religiosity

**Figure 4.**
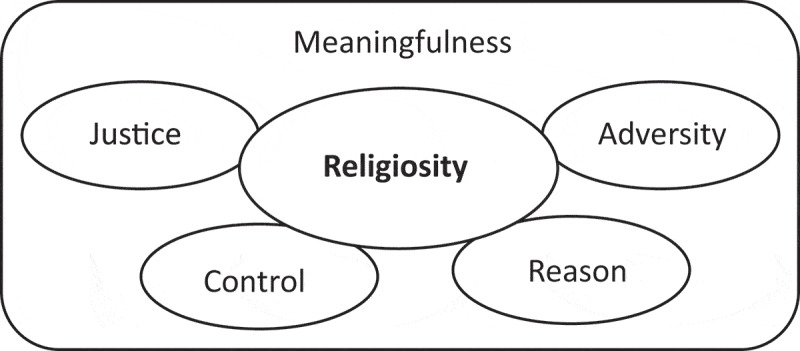
Possible relation of “Religiosity” in the meaningfulness domain.

Because of the explicit and remarkable reference to God in the described meaningfulness dimensions, we suggested an inductively found aspect of world assumptions in Sierra Leone, specifying religiosity as a subdomain of meaningfulness as depicted visually in Figure 4.

#### 3.2.4. Contrasting Sierra Leonean and Western world assumptions

By contrasting the patterns of Sierra Leonean students’ world assumptions with the ones proposed for Western societies (Janoff-Bulman, 1989), Table 4 makes the reason for renaming as well as adding certain dimensions for culture-sensitive investigations visible.

**Table 4.** Patterns of our sample’s assumptions contrasted to those of Western societies.

World Assumptions	Sierra Leone (students in Freetown)	Western societies*** (Janoff-Bulman, 1989)
**Worthiness of the Self**		
Self-Concept*	Self-orientation in a collectivistic society with high and positive assumptions in all dimensions related to the self.	Strong self-focus in an individualistic society with high and positive assumptions in all dimensions related to the self.
*Self-Worth*		
*Self-Control*		
*Luck*		
Chances/Future**		
**Character (cf. Benevolence)**		
Global, societal & communal levels*	Mixed, yet rather negative assumptions about the world and other people on all three levels.	Generally positive assumptions about the world and other people.
*Character of the world (cf. Benevolence of the world)*		
*Character of people* *(cf. Benevolence of people)*		
**Meaningfulness**		
Religiosity*	Religious beliefs are very important, they help to move through life, which is experienced as unjust, but ultimately destined by God. Destiny/life is thought to be influenceable by right (religious) behavior. Adversity is seen as usual and a part of life.	Religious beliefs are not important in life, which is experienced as just, and controllable by behavior. Adversity is seen as unusual.
*Justice*		
*Controllability*		
*Reason (cf. Randomness)*		
Adversity**		

*additional subdomain; **additional dimension; ***proposed pattern according to literature.

#### 3.2.5. Additional expert’s opinion: impact of trauma on world assumptions

Asked about the general impact of a trauma on people’s assumptions, expert 2 stated that it:

… is shocking, devastating, unanticipated. It is shattering … they think it is unexpected, they hope that it is not going to happen. They believe in God, and if it does happen it’s because God designed it. … But wherever you go you see people experiencing flashbacks. The trauma comes back once in a while.

### Expert 1 further mentioned possible assumptions to be shattered:

And then they also start to question: “This church community that I saw before as a family, a loving people, are they really loving people? … Is this even the right place to be? Am I in the right place?” Some may even ask: “Does God exist?”

### 3.3. Quantitative findings

#### 3.3.1. Descriptive statistics

The mean total scores in the sample (*N* = 280) were 28.35 (*SD* = 8.46) for the CBI (range 5–45, possible range 0–45) and 22.54 (*SD* = 6.35) for the CES (range 9–35, possible range 7–35). Table 5 presents detailed descriptive statistics for the CBI and CES items in our quantitative sample and additionally provides the ones from the qualitative participants for contextualization.

**Table 5.** Mean item scores and standard deviations of applied measurement items.

Scale/item	*N* = 280		*n* = 16	
	M	SD	M	SD
CBI total	**3.15**	0.94	**3.39**	0.71
CBI_1: Because of the event, I seriously examined the degree to which I believe things that happen to people are fair.	**2.27**	1.81	**2.37**	1.89
CBI_2: Because of the event, I seriously examined the degree to which I believe things that happen to people are controllable.	**2.63**	1.67	**2.93**	1.87
CBI_3: Because of the event, I seriously examined my assumptions concerning why other people think and behave the way they do.	**2.96**	1.51	**3.00**	1.59
CBI_4: Because of the event, I seriously examined my beliefs about my relationships with other people.	**3.09**	1.58	**3.50**	1.86
CBI_5: Because of the event, I seriously examined my beliefs about my own abilities, strengths and weaknesses.	**3.25**	1.64	**3.70**	1.24
CBI_6: Because of the event, I seriously examined my beliefs about my expectations for my future.	**3.56**	1.56	**4.06**	1.00
CBI_7: Because of the event, I seriously examined my beliefs about the meaning of my life.	**3.50**	1.56	**3.81**	1.64
CBI_8: Because of the event, I seriously examined my spiritual or religious beliefs.	**3.55**	1.53	**3.31**	1.96
CBI_9: Because of the event, I seriously examined my beliefs about my own value or worth as a person.	**3.56**	1.63	**3.75**	1.39
CES total	**3.22**	0.91	**3.57**	0.96
CES_1: I feel that this event has become part of my identity.	**2.81**	1.58	**3.12**	1.59
CES_2: This event has become a reference point for the way I understand myself and the world.	**3.53**	1.30	**3.93**	1.10
CES_3: I feel that this event has become a central part of my life story.	**3.38**	1.47	**3.87**	1.41
CES_4: This event has influenced the way I think and feel about other experiences.	**3.27**	1.35	**3.54**	1.25
CES_5: This event permanently changed my life.	**3.13**	1.51	**3.44**	1.63
CES_6: I often think about the effects this event will have on my future.	**3.17**	1.36	**3.50**	1.32
CES_7: This event was a turning point in my life.	**3.25**	1.47	**3.56**	1.59

CBI = Core Beliefs Inventory (possible range 0–5); CES = Centrality of Event Scale (possible range 1–5)

The independent sample t-tests showed no significant differences in CBI and CES totals, neither between men and women nor between Muslims and Christians. Yet, considering each item separately, a significant difference was found in CES_1 between men and women *t* (278) = 2.15, *p* = .032 (*M* male = 3.00, *M* female = 2.60) and in CBI_7 between Muslims and Christians *t* (276) = −2.14, *p* = .033 (*M* Muslim = 3.29, *M* Christian = 3.69).

#### 3.3.2. Correlations

The Pearson product-moment correlations revealed that CES and CBI total scores correlated positively in our quantitative sample (.402**, *p* < .01). Age did not significantly relate to the total scores of CBI and CES but the elapsed time since the traumatic event correlated positively with the totals of CBI .119* (*p* < .05) and CES .206** (*p* < .01).

## 4. Discussion

The primary objective of this mixed methods study was to qualitatively explore the world assumptions of Sierra Leonean students and secondarily, to quantitatively gain initial insights into the applicability and structure of the “Shattered Assumptions Theory” (Janoff-Bulman, 1992) in this West African country. Because the two strands of the embedded design addressed different temporal perspectives—current beliefs in the qualitative strand and retrospective assessments in the quantitative strand—full analytical integration is limited. Directly aligning past-oriented item scores with present-day narratives would require inferring unmeasured belief changes over time, making such connections speculative. Nevertheless, we highlight and interpret several notable points of convergence and divergence. Findings from both strands are consolidated and discussed thematically, organized by the core domains and themes.

Because earlier scholarship has documented distinct self-concepts in collectivistic societies—particularly in Sierra Leone (Winter et al., 2016)—we began our examination of self-related assumptions by investigating the self-concept of our Sierra Leonean student sample. The experts noted that the self in a collectivistic Sierra Leonean society is influenced by close relationships, marked by interdependence driven by poverty and resource scarcity, yet emphasized the importance of and orientation towards the individual self. Our results indicate that the extent of interdependence and the position of the self in collectivistic societies exist on a continuum rather than a fixed proportionality. One expert suggested that the term “collectivism” might be a disguise for reality and emphasized the prevalence of “individualism” in Freetown compared to rural areas. Consequently, our student sample in Freetown, with internet access and engagement in a Western-influenced education system, may exhibit at least some cognitive orientation towards “the self” typically attributed to Western cultures (Bracken, 1998). This group may not align entirely with traditional collectivistic norms, where the self is primarily centred on the extended family (Hobfoll et al., 2011).

We found high levels of assumptions about the self, including self-worth, self-control, and luck, throughout our sample. In the pre-established facet concerning life chances and future outlook, interviewees consistently reported a positive attitude. The experts highlighted that our student participants hold a comparatively privileged status in Sierra Leonean society, often stemming from better-off families, who support their education. One expert described Sierra Leonean people as strong and resilient, having endured atrocities such as an 11-year civil war and a two-year Ebola epidemic. As previously found in Sierra Leone, people had even reported growth stemming from adversity (Exenberger et al., 2022; Steidl et al., 2024). However, whether the general or less affluent inhabitants of Sierra Leone share this positive outlook on themselves, their chances, and their future or perceive themselves as fortunate, as our student sample did, remains questionable and requires further investigation.

In the quantitative sample the means of the referred CBI items (5, 6 & 9) and CES item 6 indicated that a traumatic event led participants to question their own self-worth and expectations for their future. For Sierra Leonean students, who are engaged in escaping poverty in a future-oriented way (a source of hope, self-efficacy and self-esteem), a traumatic event may have challenged this plan and source. Various scenarios, such as extended college closures during the Ebola and Covid-19 outbreaks or after a violent riot before the 2021 presidential election, might have put the continuation of students’ education at risk, which their future and sense of self-worth depend on. Future research should concentrate on the scope and effects of traumatic experiences in Sierra Leone. However, concerning the positive assumptions about the self in our sample after experiencing a traumatic event, it is plausible that the previously affected assumptions were restored, given that all students were actively engaged in their education. The majority reported a positive outlook on themselves and their future during the study.

Generally, “character of the world and people”, as we renamed the benevolence domain of Janoff-Bulman (1989) to reduce biases, contained a range of contrasts. The answers were furthermore related to different levels, with participants referring their statements to a global, societal, and/or communal context. On the global and societal levels, a mixed but rather negative outlook on the world and its people as well as on Sierra Leone and its inhabitants was reported. None of the participants considered the world and/or Sierra Leone to be a safe place, which is reasonable in a country described as a “hazardous place” by one expert. The ambiguous outlook on other people on the societal level may stem from a general lack of interpersonal trust in Sierra Leone, as explained historically by an expert. Betrayal among people during the civil war contributed to this mistrust, which persisted despite political and cultural efforts to “forgive and forget”. Fundamental forgiveness in Sierra Leone has been hard to achieve (Brown, 2013). On the communal level, only some participants reported to have trust in their close ones, while more denied it. The experts emphasized that trust in close relationships is also not complete. Our findings indicate that the levels (i.e., global, societal or communal) should always be specified when investigating base rate notions of how individuals perceive “the world” and “other people”. Just asking general questions does not align with the scope of various possible facets. In each culture historical, political, and societal contexts may distinctively shape a person’s assumptions of “character of the world and people” across different levels.

The CBI items (3 & 4), asking if a traumatic event led participants to question assumptions about other people, were indicated to a moderate degree. While item 3 refers to people in general, item 4 focuses on relationships with close ones. Depending on the role other people played in it, a traumatic experience may have intensified a pre-existing mistrust or challenged a pre-existing trust. As the reported traumatic events varied in nature and complexity, from human-induced events (rape, sexual violence, war) to nature-induced events (diseases and natural disasters), understanding the relationship between specific traumatic events and their impact on interpersonal dimensions of world assumptions requires further research.

Regarding the first meaningfulness dimension, about two-thirds of the qualitative sample reported that they did not experience the world or Sierra Leone as just or fair. One expert noted that only the privileged experience the world as just in Sierra Leone, primarily referring to politicians or influential people who have the chance to secure privileges for themselves. While students, who mostly come from the middle class, are not as socially advantaged as this view may imply, our results demonstrate that they do not perceive Sierra Leone as a just place, where good people necessarily experience good outcomes. Accordingly, the fact that the CBI item 1 (asking if the adverse event led the participant to seriously examine the degree to which things that happen to people are fair), was reported to the lowest degree, suggests that a belief in a just world (Lerner, 1970) may not apply to most Sierra Leoneans and may be independent of experiencing a personal traumatic event.

Even though the world has been experienced as unjust, more than half of the participants in our study expressed a belief in their ability to control or influence what happens to them through the right actions, behaviour, and mindset. On the one hand, this finding challenges Laungani’s (1997) assertion that a sense of controllability is primarily applicable to Western worldviews. On the other hand, it somewhat contradicts the findings of the third meaningfulness dimension “reason”, as we renamed it. While some attributed reasons to behaviour, over half of the participants attributed the ultimate reason for events to God or destiny and clearly rejected the idea of randomness. But only a few participants explicitly considered destiny to be influenceable. One interviewee highlighted a tension between religious and educational perspectives, suggesting that, despite exposure to both influences, religious beliefs may still hold stronger than rational ones among students. This might indicate that individuals without an academic background or non-students in Sierra Leone may not attribute events to behaviour to the same extent as students. One interpretation is that participants reported a sense of controllability because both education and religion emphasize the influenceability of events, albeit through different mechanisms. In education, it may be viewed as an action-outcome contingency while, in religion, the emphasis might be on the importance of the right religious behaviour, such as prayer and worship.

Although the corresponding CBI items for meaningfulness, including item 2 (examining beliefs about the controllability of events), indicated a lower impact compared to other CBI items, they still showed that a traumatic event influenced the feeling of controllability among students. Unfortunately, the study’s design did not allow us to determine whether the sense of controllability was stronger before the traumatic event occurred than reported at time of study participation.

In general, traumatic events clearly influenced the participants’ assumptions about meaningfulness, as reflected in CBI item 7 (examining beliefs about the meaning of life). Notably, the only significant difference between Muslims and Christians was found in this item, with Christians reporting a higher impact. This outcome may be related to the statement of one expert on the potential differences in how Muslims and Christians generally attribute events to destiny or God’s will. According to our results, Muslims in Sierra Leone may exhibit a stronger acceptance of destiny, independent of experiencing a traumatic event. However, they also question the meaning of life to some extent when faced with trauma.

Throughout the three dimensions of meaningfulness, a salient or latent connection to spiritual and existential beliefs was identified. Given the omnipresence of religion in Sierra Leone, where God is regarded as the ultimate origin of and cause for everything (Conteh, 2011; Winter et al., 2016), a subdomain called “Religiosity” was proposed to better understand world assumptions, especially in the context of meaningfulness in Sierra Leone. The importance of religion was also reflected by the high rate of active believers in the samples (more than 90%).

Similar to the introduction of the “principle of a metaphysical plan” in the study with unaccompanied refugee minors from Syria and Afghanistan in Germany (Gottschald & Sierau, 2020), we found the consideration of a spiritual context necessary to supplement Janoff-Bulman’s model in Sierra Leone. Unlike the study with participants from the Middle East, hence an Islamic background, our findings suggest that this additional subdomain is applicable to Christians and Muslims, at least in a Sierra Leonean context, with individuals from both religious backgrounds considering their beliefs to make sense of the world and events.

Qualitative exploration of participants’ attitudes towards the frequent occurrence of adversity in Sierra Leone revealed that more than half regarded adversity as part of everyday life. This finding aligns with Winter et al.’s (2016) observation that Sierra Leoneans generally do not view adversity as unusual. In contrast, people in Western societies typically experience traumatic events as unexpected and are therefore psychologically unprepared for them (Janoff-Bulman, 1992). Given that adversity and trauma are widely viewed as part of everyday life in Sierra Leone, one might have hypothesized that people there would be psychologically prepared for personal adversity. If so, a traumatic event would be unlikely to challenge their assumptive worlds or play as central a role in their identity as our sample’s CBI and CES scores suggest. Previous assertions that the understanding of trauma as a shattering experience is just a Western conceptualization (Winter et al., 2016) were contradicted.

However, in line with Wong et al.’s (2006) stress-coping model and Pargament’s (1997, 2011) theory of religious meaning-making, our data suggest that culturally embedded values—most notably religious narratives that portray hardship as an expected part of the human condition—shape both the appraisal of and response to traumatic events. These shared scripts operate on two levels. First, they offer cognitive re-framing: adversity is interpreted not as an inexplicable violation of order but as a familiar, even purposeful, life test, thereby preserving the assumptions that the world is broadly coherent and that suffering can be meaningful (Koenig, 2012; C. L, Park, 2010). Second, they supply socially sanctioned behavioural guidelines—rituals of prayer, communal lament, and mutual aid—that convert abstract beliefs into concrete coping actions and reinforce a sense of collective efficacy (Pargament, 1997; Tedeschi & Calhoun, 2004). Together, these mechanisms blunt the impact of trauma on core world assumptions, but they do not eliminate it, because reframing cannot fully offset the loss, fear, or injustice inherent in traumatic experience (Janoff-Bulman, 1992). Thus, cultural and religious meaning systems function as partial buffers, narrowing but not closing the gap between pre- and post-trauma worldviews.

Both quantitative measures (CBI and CES) were positively correlated, suggesting that the higher the impact of a traumatic event on core beliefs is, the more it is considered relevant to one’s life story. Additionally, CBI and CES were positively correlated with elapsed time since the reported event, indicating that the further the event occurred in the past, the more it was reported to have challenged a participant’s assumptive worlds and been central to his or her personality. Previous research informed that a personal trauma initially may have a small impact that increases as time goes by if it is considered central to the life narrative (Schuler & Boals, 2016). Another possible explanation could be that our outcomes were influenced by respondents referring to the experiences of the civil war, which happened between 33 and 22 years ago and presumably was the most powerful trauma in Sierra Leone’s recent history.

The experts mentioned that Sierra Leoneans generally maintain hope and an expectation that nothing bad will happen to them even though adversity is seen as a part of life. The impact of a traumatic event may shatter this hope, leading individuals to acknowledge the event as central to their life story. However, the subsequent attribution of the adverse event to an assimilated or accommodated destiny may help individuals to make sense of the event. Participants who mentioned that destiny could be influenced to some extent (e.g., by praying) may reflect this hope. The experts suggested that questioning religious beliefs could be a result of the shattered hope in God or destiny to provide good fortune, possibly leading to questioning the existence of God altogether. Accordingly, CBI item 8 (examining spiritual or religious beliefs) was one of the highest reported challenges after a traumatic event, indicating a substantial impact on religious beliefs and, consequently, on meaningfulness. However, participants, according to their statements at the time of participating in the study, may have restored, whether assimilated or accommodated, their assumptive worlds regarding their religion, particularly in the context of meaningfulness (i.e., destiny). Yet, it is possible that pre-trauma assumptions about spiritual beliefs and destiny were more promising than post-trauma assumptions. The central role of religion in dealing with, responding to, and overcoming trauma in Sierra Leone calls for further investigation.

To sum up, our findings regarding the Sierra Leonean assumptive worlds suggest that individuals who live in rather unstable, insecure, and adverse circumstances, orientate themselves towards the areas which may be perceived as controllable. Positive assumptions about oneself might help to cope with and move through harsh living conditions and unpredictable external factors. Above all, people seem to maintain their positivity and hope by trusting in God. Although traumatic events may threaten this hope and these positive self-views, religiosity might play a key role in overcoming the aftermath, stabilizing and restructuring one’s positive fundamental beliefs, and accepting as well as managing the negative aspects of the experience (García et al., 2017; Gottschald & Sierau, 2020; Pargament, 1997, 2011; Wong et al., 2006). In Sierra Leone and possibly in other countries and cultures facing a similar socio-economic situation, spiritual beliefs and religious coping may even facilitate growing out of adversity.

### 4.1. Clinical implications

In Sierra Leone, a civil war, epidemics, and entrenched corruption have eroded both public and interpersonal trust, making social reconstruction imperative. Culturally grounded initiatives offer promising avenues for rebuilding trust: Restorative-justice rituals like *Fambul Tok* publicly enact apology and accountability and help to mend relationships and reaffirm communal values (A. S. J. Park, 2010). Community-based dialogue circles—particularly within religious and traditional structures—can foster shared values, reciprocity, and transparency. Faith communities, known for interreligious cooperation in Sierra Leone, provide trusted spaces for rebuilding social cohesion (Graybill, 2010). Psychological interventions must align with these local traditions, emphasizing mutual aid and fairness to effectively rebuild trust.

School-based civics programmes that combine anti-corruption education with participatory decision-making help to reinforce young people’s trust in institutions and strengthen democratic engagement (Schulz, 2024). However, such psychosocial gains remain fragile without parallel progress towards transparent and accountable governance as systemic corruption continues to erode civic confidence and weaken democratic institutions (Transparency International, 2024).

For global-mental-health practice—particularly in collectivist contexts marked by collective trauma and where communal worldviews shape individual well-being—three principles follow: First, interventions should offer concrete experiences of procedural fairness, not merely teach individual coping skills. Second, programmes must be delivered through indigenous social structures—schools, faith communities, traditional courts—to ensure cultural resonance and sustainability. Third, their impact should be evaluated with locally validated measures of belief-change and social trust to ensure empirical accountability and enable cross-cultural comparisons (Haroz et al., 2020). Applied together, these steps weave personal recovery into the broader fabric of community legitimacy.

### 4.2. Limitations

It is essential to emphasize that our findings are specific to a sample of tertiary education students in an urban Sierra Leonean context (Freetown). To generalize our findings concerning other segments of Sierra Leonean society or different regions of the country is restrictive. Census data indicate that as of 2015 (the latest publicly available estimate) only 17.1% of Western Area Urban residents (i.e., greater Freetown) had completed or were enrolled in tertiary education (Statistics Sierra Leone, 2017). Moreover, while city dwellers generally benefit from superior infrastructure, services, schooling, and economic diversification, rural Sierra Leoneans remain heavily reliant on agriculture and therefore endure higher poverty, poorer health outcomes, and fewer service networks (Seforall.org, n.d.; Sserwanja et al., 2021; Trade.gov, 2024; World Bank, 2024).

A potential limitation arises from the interview setting, where the participants’ responses to interview questions about their assumptions about themselves and related aspects may have been influenced by social desirability. Despite the first author’s efforts to maintain neutrality during face-to-face interviews, the unusual interview situation and the presence of a foreign researcher might have had an impact on the participants’ disclosures.

Our samples reported of a large diversity in terms of traumatic events, their duration and the time passed by since then. These circumstances limit the conclusions from the impact of a specifc event on certain dimensions of world assumptions. Furthermore, future research should adopt longitudinal, multi-wave designs that track core beliefs over time, allowing clearer inference on how distinct traumatic experiences shape, erode, or reinforce fundamental worldviews. It is important to acknowledge that the understanding of a DSM-5 Criterion A traumatic event might not fully align with Sierra Leonean perceptions of such an event. Future research should delve into the Sierra Leonean perspectives on trauma, including sociocultural, historical, and political dimensions, to provide a more comprehensive understanding of responses to and recovery from traumatic experiences in the country.

## 5. Conclusion

We could add scientific evidence indicating that certain domains of world assumptions as proposed by Janoff-Bulman (1989) may in fact have universal aspects (Splevins et al., 2010), while acknowledging that the content of these assumptions can vary across different cultures (e.g., Laungani, 1997). However, future studies should either adopt emic, bottom-up approaches and develop culturally grounded instruments or expand and modify existing etic scales such as the WAS (Janoff-Bulman, 1992) to thoroughly capture peoples’ core beliefs. Building on our findings, we recommend to additionally focus on diverse conceptualizations of the self and to explore assumptions related to personal expectations concerning the future, attitudes towards adversity, and the role of religion. To reduce biases in culturally sensitive approaches, it might be beneficial to use neutral terms like “character” instead of “benevolence” and more open ones such as “reason” instead of “randomness” to address respective domains and dimensions of world assumptions. To examine peoples’ assumptions about the “Character of the world and people” we further propose specifying questions for our introductory subdomain “Global, societal & communal levels”, because an individual’s assumptions may vary across these levels.

Our findings also affirm the applicability of the “Shattered Assumptions Theory” in the context of Sierra Leone. However, by investigating the structure of the theory, we think that the term “shattered” may be too strong for certain facets, as a challenge to a belief, at least in West-Africa and possibly beyond, does not always imply a complete breakdown but may involve questioning and deterioration. Therefore, a more nuanced, flexible, and culturally differentiated conceptualization of the theory is essential for covering cross-cultural examinations and applications in studies related to trauma or PTG.

Furthermore, our results highlight the substantial role of personal trauma in the life story and identity of participants, regardless of the acknowledgement that adversity is part of life in Sierra Leone. Beyond the immediate impact and suffering, a personal traumatic experience may serve as a reference to and explanation of subsequent choices, behaviour, and principles of the individual (Berntsen & Rubin, 2006). These findings contribute to a better understanding of past reports of PTG in Sierra Leone (Exenberger et al., 2022; Steidl et al., 2024) and lay the foundation for future investigations into trauma-related research in West Africa.

## Data Availability

Data is not available online. Readers who wish to gain access can request by contacting Andreas Steidl (andreas.steidl@posteo.at), which would then be subject to ethical approval.
